# Depression and anxiety symptoms post-stroke/TIA: prevalence and associations in cross-sectional data from a regional stroke registry

**DOI:** 10.1186/s12883-014-0198-8

**Published:** 2014-10-01

**Authors:** Niall M Broomfield, Terence J Quinn, Azmil H Abdul-Rahim, Matthew R Walters, Jonathan J Evans

**Affiliations:** Institute of Cardiovascular and Medical Sciences, University of Glasgow, Glasgow, UK; Rehabilitation Assessment Directorate, NHS Greater Glasgow and Clyde, Glasgow, UK; Institute of Health and Wellbeing, University of Glasgow, Glasgow, UK

**Keywords:** Mood, Stroke, TIA, Anxiety, Depression, Prevalence

## Abstract

**Background:**

Mood disorders are commonly seen in those with cerebrovascular disease. Literature to-date has tended to focus on depression and on patients with stroke, with relatively little known about post-stroke anxiety or mood disorder in those with transient ischaemic attack (TIA). We aimed to describe prevalence of depression and anxiety symptoms in stroke and TIA cohorts and to explore association with clinical and socio-demographic factors.

**Methods:**

We used a city wide primary care stroke registry (Glasgow Local Enhanced Service for Stroke - LES). All community dwelling stroke-survivors were included. We described cross-sectional prevalence of depression and anxiety symptoms using the Hospital Anxiety and Depression Scale (HADS). Data on clinical and demographic details was collected and univariable and multivariable analyses performed to describe associations with HADS scores. We examined those with a diagnosis of ‘stroke’ and ‘TIA’ as separate cohorts.

**Results:**

From 13,283 potentially eligible stroke patients in the registry, we had full HADS data on 4,079. Of the 3,584 potentially eligible TIA patients, we had full HADS data on 1,247 patients. Across the stroke cohort, 1181 (29%) had HADS anxiety scores suggestive of probable or possible anxiety; 993 (24%) for depression. For TIA patients, 361 (29%) had anxiety and 254 (21%) had depression. Independent predictors of both depression and anxiety symptoms were female sex, younger age and higher socioeconomic deprivation score (all p < 0.001).

**Conclusion:**

Using HADS, we found a high prevalence of anxiety and depression symptoms in a community-based cohort of patients with cerebrovascular disease.

**Electronic supplementary material:**

The online version of this article (doi:10.1186/s12883-014-0198-8) contains supplementary material, which is available to authorized users.

## Background

Mood disorders are common in stroke-survivor cohorts and are associated with increased morbidity and mortality. Meta-analyses of point-prevalence rates suggest one third of stroke-survivors develop post-stroke depression and one quarter develop post-stroke anxiety [1,2]. More than half of stroke survivors will be affected by depression at some point [3]. These summary data are important and strongly suggestive of a high stable prevalence of post-stroke mood disorder, but meta-analyses are limited by all the caveats that accompany data pooled from various studies and populations.

Stroke-survivor populations are heterogenous and there is still value in describing contemporary patterns of post-stroke mood problems in a suitably large population. A group who are not well represented in the available literature are those whose index event would be classified as a Transient Ischaemic Attack (TIA) or “minor stroke”. Although the evidence would suggest that TIA is associated with depression, the literature is characterised by studies of only modest sample size and is far from definitive [4-8]. No large study has examined mood problems in both stroke and TIA samples, using an identical assessment procedure, to compare. Further, we are not aware of any suitably large studies of anxiety incidence or prevalence following TIA. It seems plausible that the psychological effects of TIA may differ from stroke and a focussed analysis of mood disorder in TIA patients seems warranted.

Despite their prevalence, post-stroke mood disorders are relatively under researched compared to stroke related physical disabilities. A better understanding of the risk factors for depression and anxiety following stroke and TIA could help inform research and target interventions. Of particular interest are the effects of age; socioeconomic deprivation (SED); smoking and co-morbidity on mood disorder. We know that these factors are associated with mood disorder in unselected non-stroke cohorts [9-12] and these variables are also risk factors for stroke itself [13].

A robust population level assessment of post-stroke mood disorder, that describes association with important socio-demographic variables and includes TIA or minor stroke, could provide useful data for clinical practice and service planning. We used a stroke specific, city-wide clinical registry to describe patterns and associations of post stroke depression and anxiety.

Our aims were:

- To describe point prevalence of depression and anxiety symptoms in a large cohort of urban, community dwelling stroke-survivors.
- To perform a subgroup analysis to describe depression and anxiety symptoms in those whose index event was classified as “TIA”.
- To describe the association of potential post stroke depression and anxiety with clinical and socio-demographic variables.

## Methods

We present a cross-sectional analysis of a city-wide primary care administered data resource. Conduct and reporting of all analyses was in accordance with Strengthening the Reporting of Observational Studies in Epidemiology (STROBE) guidelines [14].

### Setting

Greater Glasgow and Clyde Health Board (GG&C) provides services to a population of 1.2 million people in Glasgow, United Kingdom. Glasgow is typical of urban settings in industrialised nations, albeit with high levels of cardiovascular disease and socio-economic deprivation. Annual admissions to secondary care with stroke are around 3,000. For the calendar year of 2012–13, GG&C had 15,524 people registered with a primary care practice who were recorded as having had previous stroke event (including TIA).

### Data source

We used the Glasgow, Local Enhanced Services (LES) database (a clinical resource) for our analyses [15]. The Glasgow LES is a contractual arrangement with primary care services, incentivising all Glasgow General Practitioners (GPs) to improve the management of exemplar chronic diseases (in the first instance these were ischaemic heart disease; heart failure and stroke). LES augments the basic patient-level data collection that is required through the General Medical Services (GMS) Quality and Outcome Framework (QOF) specification, by providing financial incentives to encourage proactive case finding, by delivering annual nurse led reviews and by managing and quality controlling centralised data storage. To ensure data quality, the LES initiative funds annual practice nurse training in assessment and data input and employs data managers to ensure validity of input data. LES funding and support is available to all GP practices in GG&C and in total 209 out of 213 practices actively participate in stroke data collection and upload.

### Participants

We identified all living stroke-survivors from the LES stroke database, using the last available calendar year with full data entry and completed quality control (year 2012–13). We excluded care-home residents or housebound subjects using LES specific read-codes.

Clinical diagnoses recorded in LES are linked to hospital discharge records and primary care registers. In the GP practices covered by the LES resource, cerebrovascular diagnoses were primarily made by stroke specialist services in secondary care with access to neuro-imaging and other supplementary investigations considered standard at the time.

Diagnoses of “stroke” and “TIA” conformed to the classical World Health Organisation (WHO) definitions. We created a data subgroup limited to those with TIA diagnosis. Where a patient had both TIA and stroke recorded they were excluded from the TIA analysis.

### Patient level data

Our primary descriptor of interest was presence of depression or anxiety symptoms. We collated data on potential anxiety and depression using Hospital Anxiety and Depression Scale (HADS) scores. HADS is a mood screening tool designed for use with physically ill patients to assess for clinical anxiety and depression [16]. HADS has been validated in stroke-survivor cohorts showing reasonable test accuracy and is one of the commonest mood measures employed in stroke clinical practice [17,18]. HADS comprises part of the routine annual LES stroke assessment; all practice staff recording LES data are trained in HADS assessment with stroke patients by a specialist stroke clinical psychology service.

HADS comprises two sub scales: HADS-anxiety (HADS-A) and HADS-depression (HADS-D). We examined individual scores for each component. Within the stroke LES, HADS data are operationalised as HADS 0–7 “normal score”; HADS 8–10 “possible caseness”; and HADS ≥11 “probable caseness”.

We also collated data on sociodemographic and clinical variables. We described age, sex and race of all included patients. We assessed socioeconomic status using Scottish Index of Multiple Deprivation (SIMD). The SIMD is assigned on the basis of residence (datazone) and incorporates domains of income, employment, health, education, geographic access to services, crime, and housing [19]. We used postcode data within the LES to assign SIMD and described data as quintiles with quintile 1 representing the most deprived area. We described rates of excessive alcohol intake (defined at practice level) and smoking (defined as any current use of cigarettes or other related products). To describe co-morbidity we collated data on presence of ischemic heart disease, heart failure, diabetes, chronic obstructive pulmonary disease (linking LES to practice level clinical diagnostic data from patients’ primary care records).

Our study was approved a priori by the West of Scotland Research Ethics Service, the local (GG&C) Caldicott Guardian and the Greater Glasgow Chronic Disease Management Overseeing Data Group.

### Statistical methods

We described the total stroke-survivor cohort and “TIA” cohort separately, grouping each cohort’s baseline characteristics by trichotomised (normal, possible, probable) HADS-A and HADS-D scores respectively. We described mean (standard deviation [SD]) or median (inter-quartile range [IQR]) for continuous variables and count (percentage) for categorical variables as appropriate. Unadjusted comparisons of individual variables by HADS grouping score-groups were conducted using ANOVA or χ^2^ test depending on the distribution and nature of the data. We recorded “significance” of univariable analysis at the conventional level (*P* < 0.05). To correct for multiple analyses we also used the sequentially rejective Bonferroni method [20], under this correction “significance” was defined as *P* < 0.005 (*significant level/number of variables*, 0.05/10 = 0.005).

We calculated odds ratios (OR) and corresponding 95 percent confidence intervals (95% CI) to express the odds of depression or anxiety. For these analyses, we defined caseness for anxiety as HADS-A score ≥8 (i.e. possible and probable cases), and caseness for depression as HADS-D score ≥8 [17]. We performed a series of univariable analyses using binary logistic regression employing dichotomized outcome measures for both anxiety and depression. We used these results to inform the choice of factors to include in the multivariable analyses. Final choice of input variables to the model were: age, sex, socio-economic deprivation (SIMD), current smoker and previous history of COPD. All analyses were undertaken using SAS version 9.2 (SAS Institute, Inc., Cary, NC, USA).

## Results

### Stroke cohort

**Figure 1:**
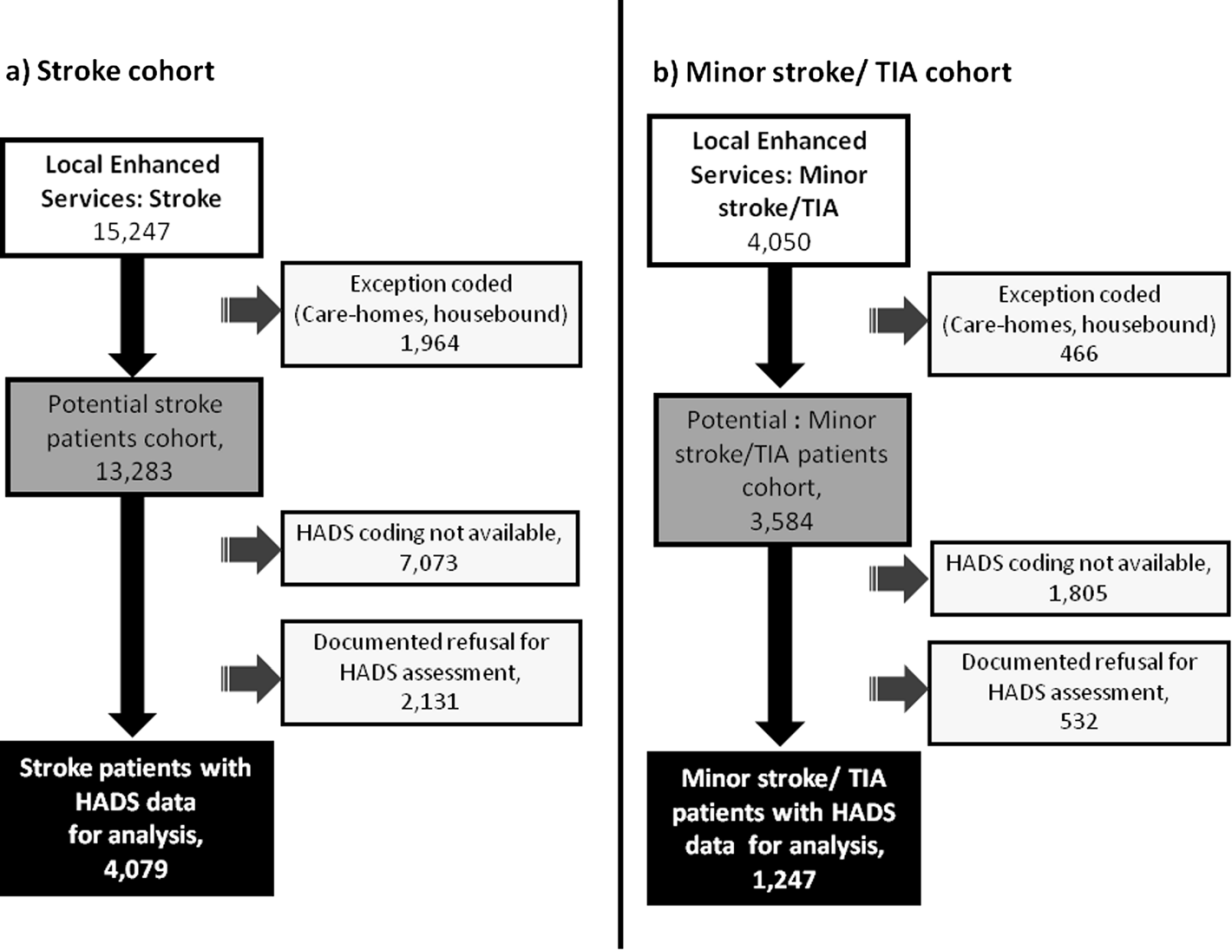
**Flow chart for selection of; a) stroke cohort and, b) minor stroke/TIA cohort; from the local enhanced service stroke database.**

The LES Stroke Database for April 2012- March 2013 contained case reviews on 15,247 stroke patients. Of the total number potentially available, 1,964 were excluded as care-home residents or housebound status. From the remaining 13,283 stroke patients; 2,131 declined to complete the HADS assessment, and 7,073 had missing HADS data; leaving a cohort with full data of 4,079 (29% of review attendees) (Figure 1a). The median age of the stroke cohort was 70.3 years (IQR: 11.3) and 2,323 persons were male (57%) (Table 1).

**Table 1:** **Baseline characteristics of stroke and TIA cohorts**

	**Cohort**				**P-value**
	**Stroke**		**TIA**		
	**(n = 4,079)**		**(n = 1,247)**		
Male	2,323	(57.0)	643	(51.6)	**<0.001**
Age; *median (IQR)*	70.3	(11.3)	70.7	(10.9)	0.178
Caucasian	3,819	(93.6)	1,167	(93.6)	0.524
Socioeconomic deprivation					**0.024**
SIMD I *(most deprived)*	1,610	(39.5)	550	(44.1)	
SIMD II	767	(18.8)	200	(16.0)	
SIMD III	483	(11.8)	145	(11.6)	
SIMD IV	447	(11.0)	141	(11.3)	
SIMD V *(least deprived)*	715	(17.5)	195	(15.6)	
Alcohol intake excessive	200	(4.9)	50	(4.0)	0.192
Current smoker	920	(22.6)	306	(24.5)	0.145
Co-morbidities					
Ischaemic heart disease	784	(19.2)	310	(24.9)	**<0.001**
Heart failure	263	(6.5)	70	(5.6)	0.287
Diabetes	1,088	(26.7)	262	(21.0)	**<0.001**
COPD	553	(13.6)	192	(24.5)	0.101
HADS; *median (IQR)*					
HADS-anxiety	4	(5)	5	(5)	0.469
HADS-depression	4	(4)	4	(5)	**0.008**

*Significant values after Bonferroni correction (P < 0.005) in bold. SIMD: Scottish Index of Multiple Deprivation-described as quintiles. IQR: inter-quartile range.*

In our stroke-survivor cohort, 604 (15%) of stroke patients were classified as definite abnormal for anxiety symptoms (HADS-A: ≥11) and 577 (14%) were classified as possible abnormal (HADS-A: 8–10); while 458 (11%) were definite abnormal and 535 (13%) were possible abnormal for depression symptoms.(Additional file 1: Table S1 and S2) 1,445 (35%) stroke patients had any mood disorder that comprises of 2,174 caseness for either anxiety or depression.

On univariable analyses, female sex; younger age; higher level of SED; smoking and presence of COPD diagnosis were all strongly associated with higher anxiety and depression scores.(Additional file 1: Figure S1 and S2) On multivariable analysis, sex, age and SED were independently associated with both anxiety and depression symptoms (Additional file 1: Table S3 and S4).

### TIA cohort

From all LES stroke entries, 4,050 case reviews were completed for patients with a clinical diagnosis of TIA and no stroke*.* Of these, 466 were excluded as care-home residents or housebound status, 532 refused the HADS assessment and 1,805 had missing HADS data. Thus, the total TIA dataset with useable data comprised 1,247 patients (Figure 1b). The TIA group had median age 70.8 years (IQR: 10.9) and 643 were male (52%). Comparison of the baseline characteristics of stroke and TIA cohorts were given in Table 1.

**Figure 2:**
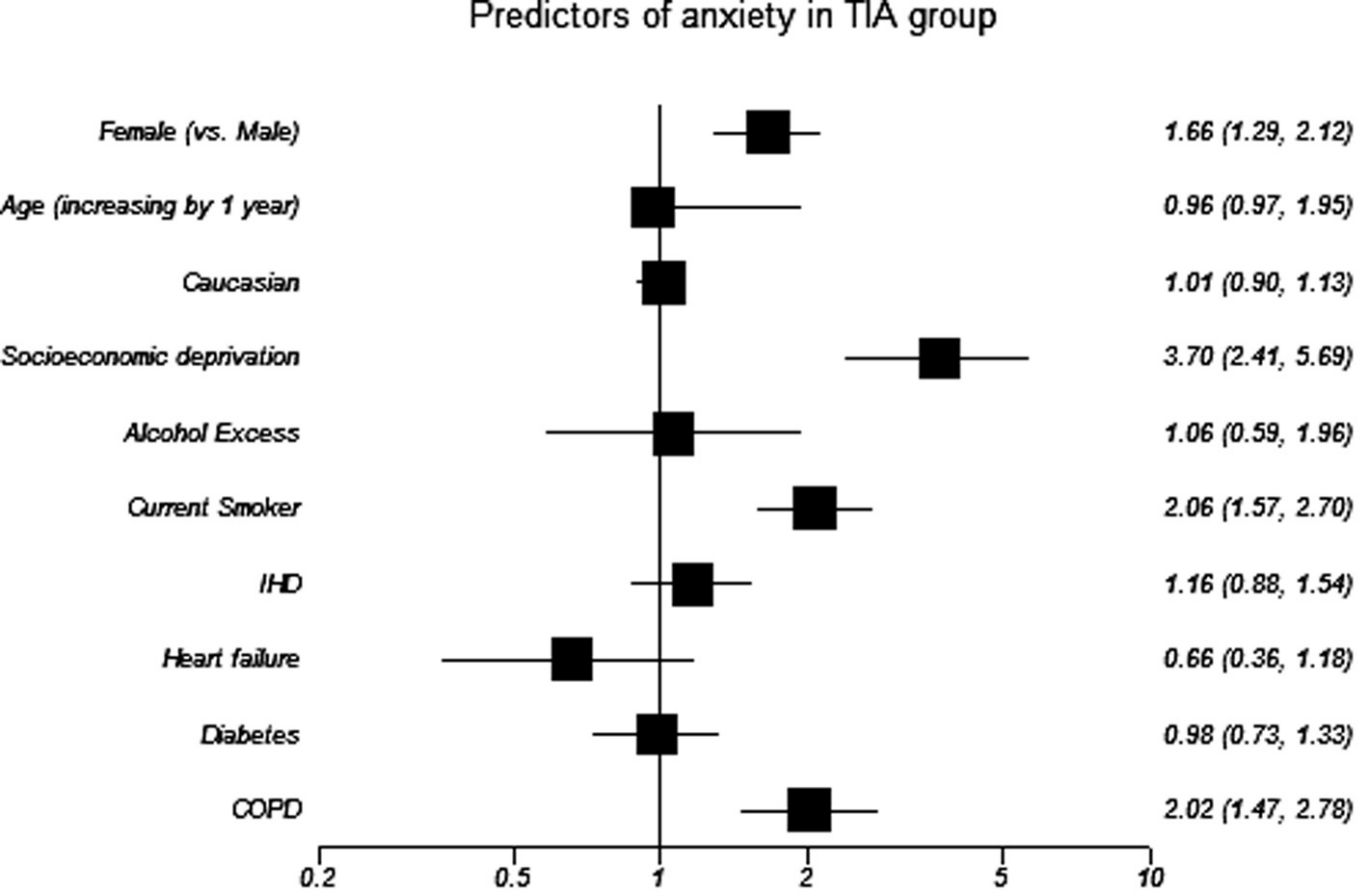
**Forrest plot shows variable’s association with caseness for anxiety in TIA cohort (unadjusted univariable analysis).** IHD: ischaemic heart diease; COPD: Chronic Obstructive Pulmonary Disease. OR and corresponding 95% CI express the odds of caseness for anxiety in univariable analysis.

**Figure 3:**
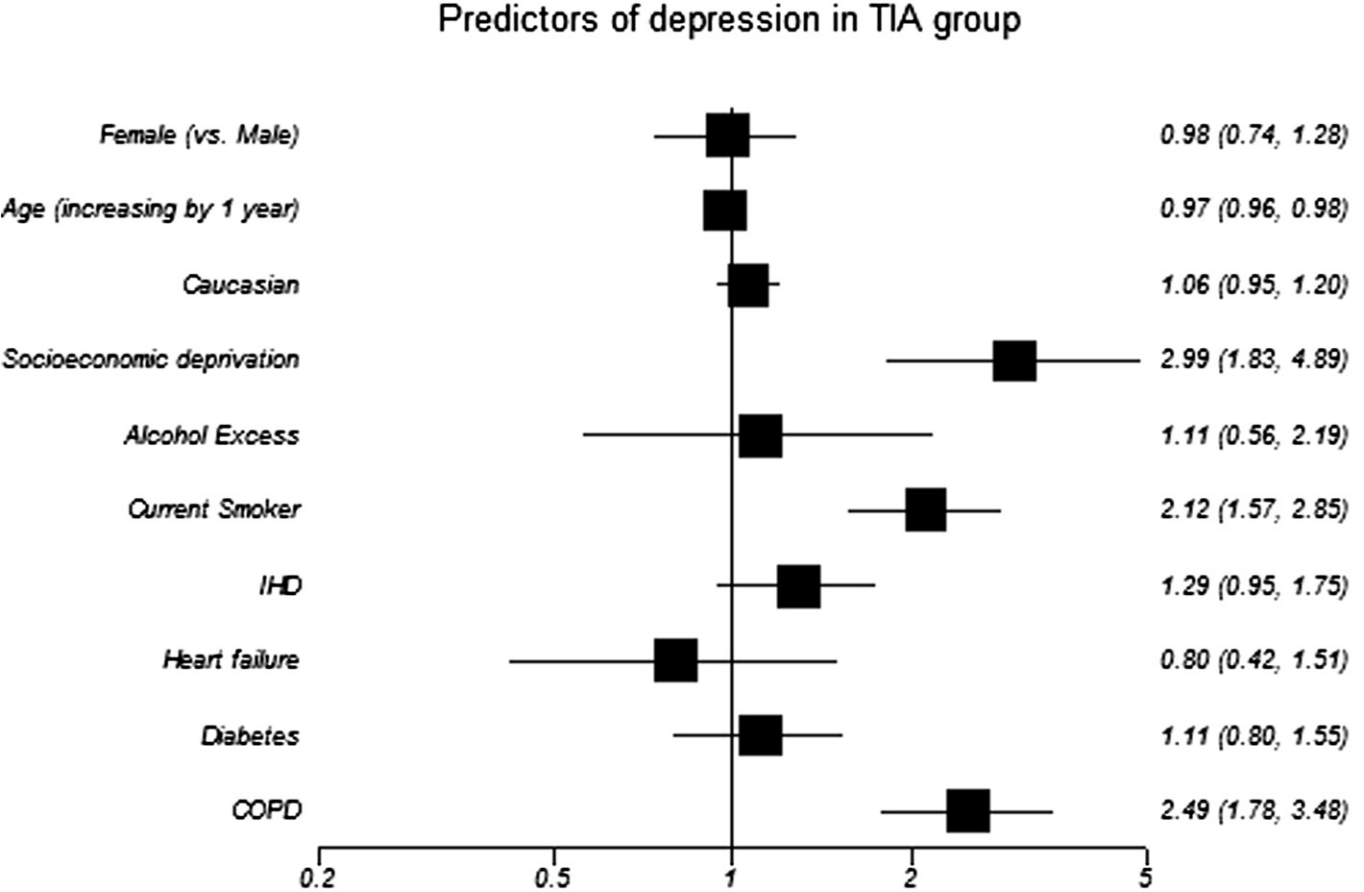
**Forrest plot shows variable’s association with caseness for depression in TIA cohort (unadjusted univariable analysis).** IHD: ischaemic heart diease; COPD: Chronic Obstructive Pulmonary Disease. OR and corresponding 95% CI express the odds of caseness for depression in univariable analysis.

In the TIA cohort, 179 (14%) were classified as definite abnormal anxiety symptoms and 182 (15%) possible abnormal; while 107 (9%) were definite abnormal for depression symptoms and 147 (12%) were possible abnormal (Tables 2 and 3) 415 (33%). TIA patients had any mood disorder that comprises of 615 caseness for either anxiety or depression. Significant univariable associations were: younger age; female sex (anxiety only); SED; smoking and COPD diagnosis (Figures 2 and 3). After multivariable analysis, all remained independently associated with anxiety and depression other than smoking (Table 4).

**Table 2:** **Baseline characteristics of TIA cohort according to HADS-anxiety scores**

	**All patients, n (%)**		**HADS anxiety scores**						**P-value**
			**0-7**		**8-10**		**≥11**		
	**(N = 1,247)**		**(n = 886)**		**(n = 182)**		**(n = 179)**		
Male	643	(51.6)	489	(55.2)	70	(38.5)	84	(47.0)	**<0.001**
Age; *median (IQR)*	70.8	(10.9)	72.3	(10.6)	69.0	(10.7)	65.0	(10.5)	**<0.001**
Caucasian	1167	(93.6)	828	(93.45)	174	(95.6)	165	(92.2)	0.166
Socioeconomic deprivation									**<0.001**
SIMD I *(most deprived)*	550	(44.1)	334	(37.7)	98	(53.9)	118	(66.0)	
SIMD II	200	(16.0)	146	(16.5)	28	(15.4)	26	(14.5)	
SIMD III	145	(11.6)	115	(13.0)	18	(9.9)	12	(6.7)	
SIMD IV	141	(11.3)	116	(13.1)	11	(6.0)	14	(7.8)	
SIMD V *(least deprived)*	195	(15.6)	166	(18.7)	22	(12.1)	7	(3.9)	
Alcohol intake excessive	50	(4.0)	35	(4.0)	7	(3.9)	8	(4.5)	0.942
Current smoker	306	(24.5)	181	(20.4)	60	(33.0)	65	(36.3)	**<0.001**
Co-morbidities									
Ischaemic heart disease	310	(24.9)	213	(24.0)	48	(26.4)	49	(24.9)	0.563
Heart failure	70	(5.6)	55	(6.2)	10	(5.5)	5	(2.8)	0.194
Diabetes	262	(21.0)	187	(21.1)	36	(19.8)	39	(21.8)	0.889
COPD	192	(15.4)	111	(12.5)	34	(18.7)	47	(26.3)	**<0.001**

*Significant values after Bonferroni correction (P < 0.005) in bold. SIMD: Scottish Index of Multiple Deprivation-described as quintiles. IQR: inter-quartile range.*

**Table 3:** **Baseline characteristics of TIA cohort according to HADS-depression scores**

	**All patients, n (%)**		**HADS depression scores**						**P-value**
			**0-7**		**8-10**		**≥11**		
	**(N = 1,247)**		**(n = 993)**		**(n = 147)**		**(n = 107)**		
Male	643	(51.6)	511	(51.5)	71	(48.3)	61	(57.0)	0.386
Age; *median (IQR)*	70.8	(10.9)	71.6	(10.7)	69.3	(11.1)	65.0	(10.6)	**<0.001**
Caucasian	1167	(93.6)	932	(93.9)	138	(93.9)	97	(90.7)	0.280
Socioeconomic deprivation									**<0.001**
SIMD I *(most deprived)*	550	(44.1)	404	(40.7)	79	(53.7)	67	(62.6)	
SIMD II	200	(16.0)	159	(16.0)	23	(15.7)	18	(16.8)	
SIMD III	145	(11.6)	120	(12.1)	15	(10.2)	10	(9.4)	
SIMD IV	141	(11.3)	124	(12.5)	8	(5.4)	9	(8.4)	
SIMD V *(least deprived)*	195	(15.6)	174	(17.5)	18	(12.2)	3	(2.8)	
Alcohol intake excessive	50	(4.0)	39	(3.9)	8	(5.4)	3	(2.8)	0.547
Current smoker	306	(24.5)	213	(21.5)	43	(29.3)	50	(46.7)	**<0.001**
Co-morbidities									
Ischaemic heart disease	310	(24.7)	237	(23.9)	45	(30.6)	28	(26.2)	0.199
Heart failure	70	(5.6)	58	(5.8)	7	(4.8)	5	(4.7)	0.788
Diabetes	262	(21.0)	205	(20.6)	40	(27.2)	17	(15.9)	0.075
COPD	192	(15.4)	125	(12.6)	30	(20.4)	37	(34.6)	**<0.001**

*Significant values after Bonferroni correction (P < 0.005) in bold. SIMD: Scottish Index of Multiple Deprivation-described as quintiles. IQR: inter-quartile range.*

**Table 4:** **Multivariable analysis of caseness for anxiety or depression in TIA cohort**

	**OR (95% ** **CI)**	**P-value**
**Caseness for anxiety**		
Female *(vs. Male)*	1.68 (1.29-2.18)	<0.001
Age *(increasing by 1 year)*	0.96 (0.95-0.97)	<0.001
Socioeconomic deprivation *(SIMD I vs SIMD V)*	2.37 (1.51-3.72)	<0.001
COPD *(vs. those not)*	1.81 (1.28-2.56)	0.001
**Caseness for depression**		
Age *(increasing by 1 year)*	0.97 (0.96-0.99)	<0.001
Socioeconomic deprivation *(SIMD I vs SIMD V)*	2.04 (1.22-3.40)	0.001
COPD *(vs. those not)*	2.17 (1.52-3.12)	<0.001

*SIMD: Scottish Index of Multiple Deprivation- described as quintiles.*

*Input covariates detailed in main manuscript.*

Given the substantial proportion with missing data, we performed post-hoc analyses comparing those with and without HADS data (declined or data missing) (Additional file 1: Table S5 for stroke patients and Additional file 1: Table S6 for TIA patients). Within the group who did not contribute HADS data, we compared those who refused HADS, with those for whom no data were recorded for other reasons, “refusers” were more likely to be male, smokers and have other co-morbidities (Additional file 1: Table S7 for stroke patients and Additional file 1: Table S8 for TIA patients).

## Discussion

To our knowledge, this is the first study to present population level data, describing post-stroke anxiety and depression symptom prevalence in stroke and TIA samples, using an identical assessment procedure (HADS), to compare. Our data add to the literature on mood disorder and stroke [21], in particular post-stroke anxiety and mood problems following TIA have received limited research attention to date and we designed our analyses to focus on those groups.

Our data suggest that depression and anxiety symptoms are common following stroke events, including TIA. Indeed, it is noteworthy that depression and anxiety levels are similar between these two patient groups. The single time point, cross-sectional data presented, does not allow us to look at temporal sequence and so we can make no inferences about causation. However, these data are in keeping with other studies that suggest stroke disease may be responsible for increased rates of mood disorder [1,2]. Certainly, the prevalence rates we describe are higher than seen in “unselected” community populations assessed with HADS (11.4% depressed) and in patients with coronary heart disease (11.8% depressed) [22,23].

As noted, we found that those with TIA had similar rates and predictors of mood disorder as those with stroke. This finding has implications for services. Many current guidelines suggest cognitive and mood screening of stroke-survivors but do not make specific comment on those with TIA. Our data would suggest that mood assessment should be similar for both groups and TIA should not be regarded as a “benign” diagnosis. We note recent work showing substantial cognitive deficits in patients with TIA and no stroke [24].

We assessed clinical and demographic associations with post-stroke mood symptoms. Ideally, through describing predictors of an outcome, services can target intervention. Mood disorder was increased in those with SED, a group who are often difficult to engage with chronic disease management. The finding of increased mood disorder in females is in keeping with patterns in the general (non-stroke) population [24,25], albeit our prevalence of disorder was considerably higher.

Within our mood disorder group will be a number of patients with psychological problems that predate the stroke event and have continued, rather than incident post stroke mood disorder. There is a complex interaction between mood and vascular disease and mood disorder may be a risk factor for vascular events [26]. We are unable to differentiate incident and prevalent mood problems using these cross sectional data. The data available do not allow us to look at time since event as a covariate and this limits interpretation. In terms of service design and access to mood intervention, perhaps the more important matter is the absolute proportion of stroke-survivors with problems rather than the timing or potential aetiology and the data are suited to this purpose.

The strength of our analysis is the large dataset, derived from a population representative of “real world” stroke survivors. We further demonstrate that the LES resources provide a substrate for describing health care and outcomes that can be used to shape practice [27]. Our inclusion of both depression and anxiety should give a better description of post-stroke mood disorder than previous studies that had a focus on depression alone; while including equivalent test data for both classical stroke and TIA allows for comparisons between these groups.

We acknowledge limitations in our study methodology. The main limitation was missing data, particularly HADS data. The missing data is unfortunate but not uncommon in large clinical registries. We offer some description of the groups with and without HADS data but recognise that fundamental differences are likely to exist between the groups and the stroke-survivors included in our analysis may not be representative. We acknowledge cognitive/communication disability may have limited participation and this may impact on prevalence of mood problems recorded. There are assessments that can be used where direct interview is not suitable, for example observational based depression and anxiety measures [28-30]. Where patients refused HADS testing, there is no simple solution to improving data capture. For those with data not recorded we note the high proportion who are non-Caucasian. It seems plausible that nurses performing reviews may feel unable to collect data in those with limited spoken English or where socio-cultural factors may impact on the significance of a mood disorder diagnosis. We will use this finding to review how we train nurses in mood assessments. The proportion with missing data had a high prevalence of our independent risk factors for mood disorder (female sex; greater deprivation) and it is possible that our data are an underrepresentation of the true burden of post stroke mood disorder.

Our mood disorder assessment metric was based on HADS. We accept that HADS is not a substitute for expert derived clinical diagnosis and that HADS has complex four-choice response format requiring intact working memory. HADS has been validated for use in stroke, albeit utility is poor in acute stroke settings [31] and has been shown to have good diagnostic accuracy for making diagnosis of depression or anxiety [17]. There remains a lack of consensus regarding optimal sub-scale cut-offs for stroke [32]. LES therefore employs standard cut points (≥8) to detect caseness, following recommendation by the scale authors and consistent with previous stroke research [33]. We were interested in “TIA” patients and looked specifically at this group. We recognise that the time based definition of TIA used in this analysis is becoming obsolete. A tissue based definition of TIA would have been preferable but was not possible in this historical cohort. As a group of patients with no residual neurology at 24 hours, our “TIA” cohort may be better described as TIA or minor stroke.

## Conclusions

In conclusion we have described a high prevalence of both depression and anxiety symptoms in a cohort of community dwelling stroke survivors. Rates of depression and anxiety symptoms were similar for TIA and stroke, suggesting that mood assessments and interventions should not be reserved for those with classical stroke only.

## Additional file

Additional file 1: **Online-only supplementary materials.**
