# Supplementary material for: Depression and anxiety symptoms post-stroke/TIA: prevalence and associations in cross-sectional data from a regional stroke registry
Source: BMC Neurol. 2014 Oct 1;14:198. doi: 10.1186/s12883-014-0198-8 (PMC4189556; doi:10.1186/s12883-014-0198-8)

**ADDITIONAL FILE: ONLINE SUPPLEMENT**

Online Supplement for manuscript entitled:

**Depression and anxiety symptoms post-stroke/TIA: Prevalence and associations in cross-sectional data from a regional stroke registry**

Authors: **NM Broomfield, TJ Quinn, AH Abdul-Rahim, MR Walters, JJ Evans.**

Supplemental Tables: 8

Supplemental Figures: 2

**SUPPLEMENTAL TABLES**

**Additional file 1: Table S1: Baseline characteristics of stroke cohort according to HADS-Anxiety scores**

|  | All patients, n(%)  (N=4,079) | | **HADS Anxiety Scores** | | | | | | P-value |
| --- | --- | --- | --- | --- | --- | --- | --- | --- | --- |
|  |  |  | 0-7  (n=2,898) | | 8-10  (n=577) | | ≥11  (n=604) | |  |
| Male | 2,323 | (57.0) | 1,770 | (61.1) | 279 | (48.4) | 274 | (45.3) | **<0.0001** |
| Age; *median (IQR)* | 70.3 | (11.3) | 71.6 | (10.8) | 69.5 | (11.3) | 64.5 | (11.5) | **<0.0001** |
| Caucasian | 3,819 | (93.6) | 2722 | (93.9) | 535 | (92.7) | 562 | (93.1) | 0.4611 |
| Socioeconomic deprivation |  |  |  |  |  |  |  |  | **<0.0001** |
| SIMD I *(most deprived)* | 1610 | (39.5) | 1017 | (35.1) | 259 | (44.9) | 334 | (55.3) |  |
| SIMD II | 767 | (18.8) | 551 | (19.0) | 106 | (18.4) | 110 | (18.2) |  |
| SIMD III | 483 | (11.8) | 343 | (11.8) | 77 | (13.3) | 63 | (10.4) |  |
| SIMD IV | 447 | (11.0) | 349 | (12.0) | 58 | (10.1) | 40 | (6.6) |  |
| SIMD V *(least deprived)* | 715 | (17.5) | 599 | (20.7) | 70 | (12.1) | 46 | (7.6) |  |
| Alcohol intake excessive | 200 | (4.9) | 149 | (5.1) | 20 | (3.5) | 31 | (5.1) | 0.2258 |
| Current smoker | 920 | (22.6) | 571 | (19.7) | 139 | (24.1) | 210 | (34.8) | **<0.0001** |
| Co-morbidities |  |  |  |  |  |  |  |  |  |
| Ischaemic Heart Disease | 784 | (19.2) | 528 | (18.2) | 130 | (22.5) | 126 | (20.9) | 0.0304 |
| Heart Failure | 263 | (6.5) | 189 | (6.5) | 33 | (5.7) | 41 | (6.8) | 0.7225 |
| Diabetes | 1,088 | (26.7) | 755 | (26.7) | 161 | (27.9) | 152 | (25.2) | 0.5612 |
| COPD | 553 | (13.6) | 355 | (12.3) | 90 | (15.6) | 108 | (17.9) | **0.0004** |

*Significant values after Bonferroni correction (P<0.005) in bold.*  *SIMD: Scottish Index of Multiple Deprivation-described as quintiles. IQR: inter-quartile range.*

**Additional file 1: Table S2: Baseline characteristics of stroke cohort according to HADS-Depression scores**

|  | All patients, n(%)  (N=4,079) | | **HADS Depression Scores** | | | | | | P-value |
| --- | --- | --- | --- | --- | --- | --- | --- | --- | --- |
|  |  |  | 0-7  (n=3,086) | | 8-10  (n=535) | | ≥11  (n=458) | |  |
| Male | 2,323 | (57.0) | 1,813 | (58.8) | 274 | (51.2) | 236 | (51.5) | **0.0002** |
| Age; *median (IQR)* | 70.3 | (11.3) | 71.4 | (10.8) | 68.3 | (12.3) | 64.9 | (11.5) | **<0.0001** |
| Caucasian | 3,819 | (93.6) | 2,895 | (93.8) | 500 | (93.5) | 424 | (92.6) | 0.2020 |
| Socioeconomic deprivation |  |  |  |  |  |  |  |  | **<0.0001** |
| SIMD I *(most deprived)* | 1,610 | (39.5) | 1,108 | (35.9) | 260 | (48.6) | 242 | (52.8) |  |
| SIMD II | 767 | (18.8) | 597 | (19.4) | 89 | (16.6) | 81 | (17.7) |  |
| SIMD III | 483 | (11.8) | 366 | (11.9) | 65 | (12.2) | 52 | (11.4) |  |
| SIMD IV | 447 | (11.0) | 364 | (11.8) | 47 | (8.8) | 36 | (7.9) |  |
| SIMD V *(least deprived)* | 715 | (17.5) | 612 | (19.8) | 68 | (12.7) | 35 | (7.6) |  |
| Alcohol intake excessive | 200 | (4.9) | 154 | (5.0) | 19 | (3.6) | 27 | (5.9) | 0.2108 |
| Current smoker | 920 | (22.6) | 605 | (19.6) | 139 | (26.0) | 176 | (38.4) | **<0.0001** |
| Co-morbidities |  |  |  |  |  |  |  |  |  |
| Ischaemic Heart Disease | 784 | (19.2) | 567 | (18.4) | 112 | (21.0) | 105 | (23.0) | 0.0390 |
| Heart Failure | 263 | (6.5) | 198 | (6.4) | 32 | (6.0) | 33 | (7.2) | 0.7284 |
| Diabetes | 1,088 | (26.7) | 807 | (26.2) | 146 | (27.3) | 135 | (29.5) | 0.3050 |
| COPD | 553 | (13.6) | 388 | (12.6) | 79 | (14.8) | 86 | (18.8) | **0.0010** |

*Significant values after Bonferroni correction (P<0.005) in bold. SIMD: Scottish Index of Multiple Deprivation-described as quintiles. IQR: inter-quartile range.*

**Additional file 1: Table S3: Multivariable analysis of caseness for anxiety in stroke cohort.**

| **Variables** | **OR (95% CI)** | **P-Value** |
| --- | --- | --- |
| Female *(vs. Male)* | 1.88 (1.63-2.17) | <0.0001 |
| Age *(increasing by 1 year)* | 0.96 (0.96-0.97) | <0.0001 |
| Socioeconomic deprivation | 2.33 (1.85-2.94) | <0.0001 |
| COPD | 1.38 (1.13-1.69) | 0.0017 |

*SIMD: Scottish Index of Multiple Deprivation- described as quintiles.*

*Input covariates detailed in main manuscript.*

**Additional file 1: Table S4: Multivariable analysis of caseness for depression in stroke cohort.**

| **Variables** | **OR (95% CI)** | **P-Value** |
| --- | --- | --- |
| Female *(vs. Male)* | 1.40 (1.20-1.62) | <0.0001 |
| Age *(increasing by 1 year)* | 0.97 (0.96-0.97) | <0.0001 |
| Socioeconomic deprivation | 2.07 (1.62-2.63) | <0.0001 |
| Current Smoker | 1.34 (1.13-1.60) | 0.0010 |

*SIMD: Scottish Index of Multiple Deprivation- described as quintiles.*

*Input covariates detailed in main manuscript.*

**Additional file 1: Table S5: Baseline characteristics of stroke patients with and without HADS data**

|  | Stroke patients in LES registry, n(%)  (N=13,283) | | **Stroke Patients** | | | | P-value |
| --- | --- | --- | --- | --- | --- | --- | --- |
|  |  |  | Patients **without** HADS data or refused  (n=9,204) | | Patients **with** HADS data  *(i.e final cohort)*  (n=4,079) | |  |
| Male | 6,969 | (52.5) | 4,646 | (50.5) | 2,323 | (57.0) | **<0.0001** |
| Age; *median (IQR)* | 70.6 | (12.9) | 70.7 | (13.5) | 70.3 | (11.3) | 0.0778 |
| Caucasian | 11,202 | (84.3) | 7,383 | (80.2) | 3,819 | (93.6) | **<0.0001** |
| Socioeconomic deprivation |  |  |  |  |  |  | **<0.0001** |
| SIMD I *(most deprived)* | 5,197 | (39.1) | 3,587 | (39.0) | 1610 | (39.5) |  |
| SIMD II | 2,587 | (19.5) | 1,820 | (19.8) | 767 | (18.8) |  |
| SIMD III | 1,756 | (13.2) | 1,273 | (13.8) | 483 | (11.8) |  |
| SIMD IV | 1,588 | (12.0) | 1,141 | (12.4) | 447 | (11.0) |  |
| SIMD V *(least deprived)* | 1,981 | (14.9) | 1,266 | (13.8) | 715 | (17.5) |  |
| Alcohol intake excessive | 375 | (2.8) | 175 | (1.9) | 200 | (4.9) | **<0.0001** |
| Current smoker | 2,382 | (17.9) | 1,462 | (15.9) | 920 | (22.6) | **<0.0001** |
| Co-morbidities |  |  |  |  |  |  |  |
| Ischaemic Heart Disease | 2,303 | (17.3) | 1,519 | (16.5) | 784 | (19.2) | **0.0001** |
| Heart Failure | 784 | (5.9) | 521 | (5.7) | 263 | (6.5) | 0.0758 |
| Diabetes | 2,767 | (20.8) | 1,679 | (18.2) | 1,088 | (26.7) | **<0.0001** |
| COPD | 1,517 | (11.4) | 964 | (10.5) | 553 | (13.6) | **<0.0001** |

*Significant values at conventional p<0.05. SIMD:Scottish Index of Multiple Deprivation-described as quintiles. IQR: inter-quartile range.*

**Additional file 1: Table S6: Baseline characteristics of TIA patients with and without HADS data**

|  | TIA patients in LES registry, n(%) (N=3,584) | | **TIA patients** | | | | P-value |
| --- | --- | --- | --- | --- | --- | --- | --- |
|  |  |  | Patients without HADS data or refused  (n=2,337) | | Patients with HADS data  *(i.e final cohort)*  (n=1,247) | |  |
| Male | 1,734 | (48.4) | 1,091 | (46.7) | 643 | (51.6) | 0.0054 |
| Age; *median (IQR)* | 71.0 | (12.4) | 71.1 | (13.1) | 70.7 | (10.9) | 0.4064 |
| Caucasian | 3,100 | (86.5) | 1,933 | (82.7) | 1167 | (93.6) | **<0.0001** |
| Socioeconomic deprivation |  |  |  |  |  |  | 0.0899 |
| SIMD I *(most deprived)* | 1,505 | (42.0) | 955 | (40.9) | 550 | (44.1) |  |
| SIMD II | 644 | (18.0) | 444 | (19.0) | 200 | (16.0) |  |
| SIMD III | 432 | (12.1) | 287 | (12.3) | 145 | (11.6) |  |
| SIMD IV | 430 | (12.0) | 289 | (12.4) | 141 | (11.3) |  |
| SIMD V *(least deprived)* | 529 | (14.8) | 334 | (14.3) | 195 | (15.6) |  |
| Alcohol intake excessive | 100 | (2.8) | 50 | (2.1) | 50 | (4.0) | **0.0012** |
| Current smoker | 707 | (19.7) | 401 | (17.2) | 306 | (24.5) | **<0.0001** |
| Co-morbidities |  |  |  |  |  |  |  |
| Ischaemic Heart Disease | 841 | (23.5) | 531 | (22.7) | 310 | (24.9) | 0.1502 |
| Heart Failure | 203 | (5.7) | 133 | (5.7) | 70 | (5.6) | 0.9238 |
| Diabetes | 612 | (17.1) | 350 | (15.0) | 262 | (21.0) | **<0.0001** |
| COPD | 487 | (14.0) | 295 | (12.6) | 192 | (24.5) | 0.0210 |

*Significant values at conventional p<0.05. SIMD:Scottish Index of Multiple Deprivation-described as quintiles. IQR: inter-quartile range.*

**Additional file 1: Table S7: Comparisons between stroke patients with missing HADS data and those who refused**

|  | Stroke patients with no HADS data, n(%)  (N=9,204) | | **Documented Refusal** | | | | P-value |
| --- | --- | --- | --- | --- | --- | --- | --- |
|  |  |  | No  (n=7,073)  **no data recorded* | | Yes  (n=2,131) | |  |
| Male | 4,646 | (50.5) | 3,520 | (49.8) | 1126 | (52.8) | **0.0129** |
| Age; *median (IQR)* | 70.7 | (13.5) | 70.7 | (14.1) | 70.6 | (11.4) | 0.8290 |
| Caucasian | 7,383 | (80.2) | 5,432 | (76.8) | 1,951 | (91.6) | **<0.0001** |
| Socioeconomic deprivation |  |  |  |  |  |  | **<0.0001** |
| SIMD I *(most deprived)* | 3,587 | (39.0) | 2,784 | (39.4) | 803 | (37.7) |  |
| SIMD II | 1,820 | (19.8) | 1,407 | (19.9) | 257 | (12.1) |  |
| SIMD III | 1,273 | (13.8) | 1,016 | (14.7) | 257 | (12.1) |  |
| SIMD IV | 1,141 | (12.4) | 879 | (12.4) | 262 | (12.3) |  |
| SIMD V *(least deprived)* | 1,266 | (13.8) | 895 | (12.7) | 371 | (17.4) |  |
| Alcohol intake excessive | 175 | (1.9) | 76 | (1.1) | 99 | (4.7) | **<0.0001** |
| Current smoker | 1,462 | (15.9) | 958 | (13.5) | 504 | (23.7) | **<0.0001** |
| Co-morbidities |  |  |  |  |  |  |  |
| Ischaemic Heart Disease | 1,519 | (16.5) | 1,100 | (15.6) | 419 | (19.7) | **<0.0001** |
| Heart Failure | 521 | (5.7) | 373 | (5.3) | 148 | (7.0) | **0.0034** |
| Diabetes | 1,679 | (18.2) | 1,130 | (16.0) | 549 | (25.8) | **<0.0001** |
| COPD | 964 | (10.5) | 693 | (9.8) | 271 | (12.7) | **0.0001** |

**Additional file 1: Table S8: Comparisons between TIA patients with missing HADS data and those who refused**

|  | TIA patients with no HADS data, n(%)  (N=2,337) | | **Documented Refusal** | | | | P-value |
| --- | --- | --- | --- | --- | --- | --- | --- |
|  |  |  | No  (n=1,805)  **no data recorded* | | Yes  (n=532) | |  |
| Male | 1,091 | (46.7) | 807 | (44.7) | 284 | (53.4) | **0.0004** |
| Age; *median (IQR)* | 71.1 | (13.1) | 71.4 | (13.6) | 70.0 | (11.1) | **0.0278** |
| Caucasian | 1,933 | (82.7) | 1933 | (79.6) | 497 | (93.4) | **<0.0001** |
| Socioeconomic deprivation |  |  |  |  |  |  | 0.0614 |
| SIMD I *(most deprived)* | 955 | (40.9) | 753 | (41.7) | 202 | (38.0) |  |
| SIMD II | 444 | (19.0) | 342 | (19.0) | 102 | (19.0) |  |
| SIMD III | 287 | (12.3) | 230 | (12.7) | 57 | (10.7) |  |
| SIMD IV | 289 | (12.4) | 215 | (11.9) | 74 | (13.9) |  |
| SIMD V *(least deprived)* | 334 | (14.3) | 241 | (13.4) | 93 | (17.5) |  |
| Alcohol intake excessive | 50 | (2.1) | 26 | (1.4) | 24 | (4.5) | **<0.0001** |
| Current smoker | 401 | (17.2) | 267 | (14.8) | 134 | (25.2) | **<0.0001** |
| Co-morbidities |  |  |  |  |  |  |  |
| Ischaemic Heart Disease | 531 | (22.7) | 410 | (22.7) | 121 | (22.7) | 0.9885 |
| Heart Failure | 133 | (5.7) | 104 | (5.8) | 29 | (5.5) | 0.7858 |
| Diabetes | 350 | (15.0) | 260 | (14.4) | 90 | (16.9) | 0.1535 |
| COPD | 295 | (12.6) | 223 | (12.4) | 72 | (13.5) | 0.4717 |

**SUPPLEMENTAL FIGURES**

**Additional file 1: Figure S1:** Forrest plot for predictors of caseness for anxiety in stroke cohort (unadjusted univariable analysis).

IHD: ischaemic heart diease; COPD: Chronic Obstructive Pulmonary Disease.

OR and corresponding 95% CI express the odds of caseness for anxiety in univariable analysis.


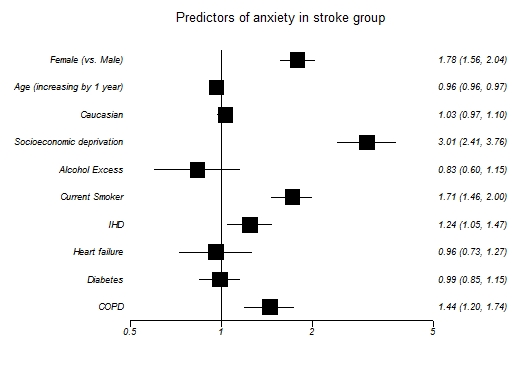


**Additional file 1: Figure S2:** Forrest plot for predictors of caseness for depression in stroke cohort (unadjusted univariable analysis).

IHD: ischaemic heart diease; COPD: Chronic Obstructive Pulmonary Disease.

OR and corresponding 95% CI express the odds of caseness for depression in univariable analysis.


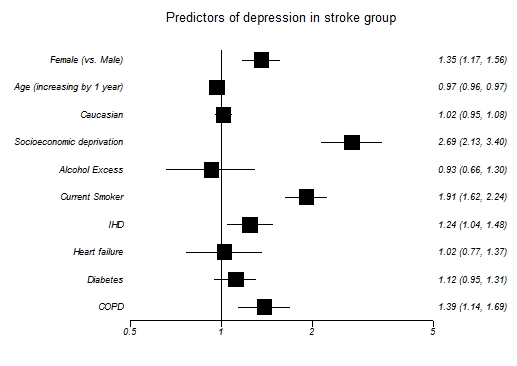

Supplement: Supplementary file 1 — Online-only supplementary materials. [file 12883_2014_198_MOESM1_ESM.docx]
